# Patch Test Results With Additions to the European Baseline Series, 2021/22—Joint European Results of the ESSCA^A^ and the EBS^B^ Working Groups of the ESCD, and the GEIDAC^C^

**DOI:** 10.1111/cod.70183

**Published:** 2026-05-14

**Authors:** Wolfgang Uter, Stephen Mark Wilkinson, Andrea Bauer, Leopoldo Borrego, Richard Brans, Timo Buhl, Heinrich Dickel, Rosella Gallo, Ana Giménez Arnau, Margarida Gonçalo, Dimitra Koumaki, Alexander A. Navarini, Cataldo Patruno, Györgyi Pónyai, Thomas Rustemeyer, Radoslaw Spiewak, Luca Stingeni, Skaidra Valiukevičienė, Marie‐L. A. Schuttelaar, Olivier Aerts, the ESSCA, EBS, ESCD working groups, the GEIDAC, Birger Kränke, Ella Dendooven, Dagmar Simon, Philip Spring, Ulrike Beiteke, Cecilia Dietrich, Sibylle Schliemann, Detlef Becker, Swen Malte John, Nicola Wagner, Elke Weisshaar, Stamatis Gregoriou, Maddalena Napolitano, Philippa Cousen, Graham A. Johnston, Juan Francisco Silvestre Salvador, José Manuel Carrascosa Carrillo, Mónica Munera Campos, Esther Serra Baldrich, Gemma Melé‐Ninot, Cristina Barrabés Torrella, David Pesqué, Ana María Giménez Arnau, María Antonia Pastor Nieto, Francisco Navarro Triviño, Leopoldo Borrego, Inmaculada Ruiz Gonzales, Enrique Gómez de la Fuente, Susana Córdoba Guijarro, Francisco Javier Ortiz de Frutos, Fátima Tous Romero, Tatiana Sanz Sánchez, Pablo Chicharro Manso, Marta Andreu Barasoain, Marta Elosua González, Araceli Sánchez Gilo, Paloma Sánchez‐Pedreño Guillén, Pedro Mercader‐García, Carmen Paredes Suárez, María Elena Gatica Ortega, José Juan Pereyra Rodríguez, Javier Miquel Miquel, Mercedes Rodríguez Serna, Violeta Zaragoza Ninet, Ricardo González Pérez

**Affiliations:** ^1^ Department of Medical Informatics, Biometry and Epidemiology Friedrich‐Alexander‐Universität Erlangen‐Nürnberg Erlangen Germany; ^2^ Dermatology Department Leeds Teaching Hospitals NHS Trust Leeds UK; ^3^ Department of Dermatology University Hospital Carl Gustav Carus, Technical University Dresden Germany; ^4^ Complejo Hospitalario Universitario Insular Materno Infantil, Universidad de Las Palmas de Gran Canaria Las Palmas de Gran Canaria Spain; ^5^ Institute for Interdisciplinary Dermatologic Prevention and Rehabilitation (iDerm) at Osnabrück University Osnabrück Germany; ^6^ Department of Dermatology Venereology and Allergology, University Medical Center Göttingen Göttingen Germany; ^7^ Department of Dermatology, Venereology and Allergology St. Josef Hospital, University Medical Center, Ruhr University Bochum Bochum Germany; ^8^ Section of Dermatology, Department of Health Sciences University of Genoa and Ospedale Policlinico San Martino, IRCCS Genoa Italy; ^9^ Department of Dermatology, Hospital del Mar Research Institute Universitat Pompeu Fabra de Barcelona Barcelona Spain; ^10^ Department of Dermatology University Hospital, Coimbra Local Health Unit and Faculty of Medicine, University of Coimbra Coimbra Portugal; ^11^ Department of Dermatology University Hospital of Heraklion Heraklion Greece; ^12^ Department of Biomedicine University Hospital, University of Basel Basel Switzerland; ^13^ Department of Dermatology University Hospital Basel Basel Switzerland; ^14^ Department of Medicine and Health Sciences “Vincenzo Tiberio” University of Molise Campobasso Italy; ^15^ Department of Dermatology, Venerology and Dermato‐Oncology Semmelweis University Budapest Hungary; ^16^ Department of Dermatology Amsterdam University Medical Centers Amsterdam the Netherlands; ^17^ Department of Experimental Dermatology and Cosmetology Faculty of Pharmacy, Jagiellonian University Medical College Krakow Poland; ^18^ Instytut Dermatologii Krakow Poland; ^19^ Section of Dermatology, Department of Medicine and Surgery University of Perugia Perugia Italy; ^20^ Department of Skin and Venereal Diseases The Hospital of Lithuanian University of Health Sciences Kauno Klinikos Kaunas Lithuania; ^21^ Department of Dermatology University Medical Center Groningen, University of Groningen Groningen the Netherlands; ^22^ Department of Dermatology University Hospital Antwerp (UZA) and Research Group Immunology, Infla‐Med Centre of Excellence, University of Antwerp Antwerp Belgium

**Keywords:** baseline series, clinical epidemiology, contact allergy, patch testing, RRID:SCR_001905, surveillance

## Abstract

**Background:**

Allergens are periodically added to the European Baseline Series (EBS) to audit their value in consecutive patch testing.

**Objectives:**

To present results of audit allergens tested in consecutive patients in 54 departments in 13 European countries during 2021 and 2022.

**Methods:**

Anonymized or pseudonymized individual data, and partly aggregated data on patch test rest results and their clinical relevance were prospectively collected and centrally pooled and analyzed.

**Results:**

In 2021 and 2022, 18 832 patients were patch tested with the EBS, and 2860 with the TRUE Test baseline series (supplemented with some missing EBS allergens). Of these patients, 17 359 were additionally tested with one or more of the 19 audit allergen preparations examined. Apart from the terpene hydroperoxides (OOH) which yielded the highest numbers of positive patch test reactions (linalool‐OOH 1% pet.: 7.65 (7.09%–8.23%) [95% CI] and limonene‐OOH 0.3% pet. 6.78 (6.26%–7.32%)), positive reactions were most often seen to benzisothiazolinone (3.71 (3.3%–4.15%)), sodium metabisulfite (3.49 (3.14%–3.88%)), *Evernia furfuracea* (tree moss, 1.79 (1.4%–2.26%)), and decyl and lauryl glucoside, at 1.49 (1.25%–1.76%) and 1.26 (1.05%–1.51%), respectively. The other allergens yielded around 1% or fewer positive patch test reactions. Clinical relevance varied, with *Evernia furfuracea*, 2‐bromo‐2‐nitropropane‐1,3‐diol and terpene hydroperoxides considered relevant in more than 2/3 cases, while for other frequent sensitizers benzisothiazolinone and sodium metabisulfite, relevance could be confirmed in half of the cases or less often.

**Conclusions:**

The current results, along with other published data, will serve to inform decisions concerning possible revision of the EBS, considering frequency of positive reactions, their clinical relevance and the overall reaction profile of each of the allergens.

## 1 Introduction

A “baseline” series should comprise those contact allergens which are of greatest importance and relevance for the majority of patients. It is regularly used for patch in testing all patients in whom allergic contact dermatitis is suspected or should be excluded. Its composition varies in time and with geographical region, reflecting changes and differences of exposure, respectively. Following fundamental conceptual thoughts delineating the objectives of a baseline series published two decades ago [1], the criteria for inclusion have recently been revisited [2]. The 2019 version of the European Baseline Series (EBS) [3] has been used until end of 2022, followed by the 2023 version [4]. The present paper summarizes results obtained with the recommended additions and with additional allergens under scrutiny for possible inclusion into the EBS in the years 2021/22. These “audit” allergens are not (yet) included in the EBS, but regularly evaluated for EBS inclusion, or alternatively recommended to be used in a targeted way, that is, in special patch test series. Data were contributed by members of the European Surveillance System on Contact Allergies (ESSCA), a working group of the European Society of Contact Dermatitis (ESCD; https://www.escd.org), by members of the EBS taskforce of the ESCD, and, in terms of aggregated results, by the Spanish “Grupo Español de Investigación en Dermatitis de Contacto y Alergia Cutánea” (GEIDAC)/“Registro Español de Dermatitis de Contacto” (REIDAC) surveillance network described in [5].

## 2 Methods

Concerning the methodological background, the reader is referred to the parallel publication on results with the EBS obtained 2021/22 [6]. In brief, most members of above‐mentioned groups, besides EBS results, also contributed anonymized or pseudonymized individual data on additional “audit” allergens. The Spanish REIDAC network as well as one UK department (Leeds) provided aggregated (results) data, that is, the number of patients tested, the number of positive, and of doubtful reactions for the allergens within scope of this analysis. All groups adhered to the ESCD patch test guideline [7]. As with the EBS results analysis, the maximum reaction between day (D) 3 and D5 (inclusive) was used as outcome, following current ESSCA standards (see discussion in [6]). As the clinical relevance of positive patch test reactions is of particular interest when evaluating allergens as potential candidates for the EBS, the usual reluctance to use relevance information, mostly due to lack of standardized definitions and usage [8], has been set aside. Fully conscious of the “softness” of this data, the frequencies of relevance attribution as “current,” “current/occupational,” “current/nonoccupational,” “past,” “past/occupational,” and “past/nonoccupational” was descriptively examined. In this context, “possible” and even “probable” relevance had not been considered, but only “certain” relevance, where such subdivision had been possible at all—or relevance which had not been further qualified, as common in most departments' usage. If more than one concentration of an allergen is tested in a patient, clinical relevance would not differ between different concentrations; however, in case different patients (in different departments) were tested with different concentrations, clinical relevance was stratified for test concentration.

It has to be noted that concomitant reactivity could only be assessed if individual data were present for the allergens involved, which was only partially the case. Hence, the numbers of overall tested of two allergens involved may be considerably higher than the number of actually usable paired patient data sets; the effective sample size always being specified. Data management and statistical analysis have been performed with the R statistical software (https://www.r‐project.org; RRID:SCR_001905), version 4.4.x., following pertinent guidelines, including exact 95% confidence intervals (CIs) to proportions (%) [9]. For the calculation of 95% confidence intervals (CIs) to zero proportions an approximation to an exact CI was used [10]. Cross‐reactivity between contact allergens was illustrated by use of a bivariate odds ratio (OR) with accompanying 95% confidence interval (CI).

## 3 Results

Among the 22 891 patients consecutively patch tested in the 66 participating departments in 13 European countries during the reporting period 2021–22, 18 832 patients were patch tested with the EBS, and in Spain, 2702 patients with the TRUE Test (SmartPractice, Phoenix, AZ), usually supplemented with EBS allergens considered important, such as fragrance mix II or methylisothiazolinone.

Many departments furthermore tested consecutive patients with one or several, rarely all, of the 19 allergens in the scope of this publication; in total, 57 departments. Altogether 17 346 (92.1%) patients were tested with at least one, but usually several of the additional allergens, or 80.6% if those tested with the TRUE Test are additionally considered as denominator. The number of patients is shown as a breakdown for country in Table 1, along with the numbers of departments contributing from that country. The individual contribution by department and year is shown in Table S1. Population characteristics according to the MOAHLFA(P) [11] mostly correspond to those presented in the EBS results analysis of the same reporting period [6], and are shown in Table S2. Patch test results with the 19 allergens tested along the (European) baseline series are shown in Table 2.

**TABLE 1:** Country‐wise contribution of patch testing with (some of) the audit allergens considered and number of departments per country.

Country	*N* (depts.)	*N* (tested)
AT (Austria)	1	184
BE (Belgium)	1	891
CH (Switzerland)	4	1077
DE (Germany)	11	3408
ES (Spain)	26	3872
GR (Greece)	2	501
HU (Hungary)	1	509
IT (Italy)	3	1344
LT (Lithuania)	1	701
NL (The Netherlands)	2	1929
PL (Poland)	1	86
PT (Portugal)	1	524
UK (United Kingdom)	3	2320
*Total*	*57*	*17 346*

*Note:* For further detail, see Table S1.

**TABLE 2:** Patch test results (day 3 to day 5) with audit additions to the European Baseline Series in consecutive patients in the 57 active departments of the European Surveillance System on Contact Allergies (ESSCA), additional contributors from the EBS working group, and the contributing “Grupo Español de Investigación en Dermatitis de Contacto y Alergia Cutánea” (GEIDAC) members.

Allergen	Conc.	Tested	+	++/+++	?+/IR	% pos. (95% CI)
Sodium metabisulfite	1.0	9703	246	93	83	3.49 (3.14–3.88)
2‐Bromo‐2‐nitropropane‐1,3‐diol	0.5	6441	24	10	11	0.53 (0.37–0.74)
Diazolidinyl urea	2.0	8972	42	10	12	0.58 (0.43–0.76)
Imidazolidinyl urea	2.0	9618	47	10	27	0.59 (0.45–0.77)
Compositae mix II^a^	2.5	5147	51	21	27	1.40 (1.10–1.76)
Compositae mix II^a^	5.0	4755	40	26	17	1.39 (1.08–1.76)
Linalool hydroperoxides	1.0	8566	522	133	259	7.65 (7.09–8.23)
Linalool hydroperoxides	0.5	9091	367	67	163	4.77 (4.34–5.23)
Limonene hydroperoxides	0.3	8911	480	124	197	6.78 (6.26–7.32)
Limonene hydroperoxides	0.2	7046	308	73	130	5.41 (4.89–5.96)
Benzisothiazolinone	0.1	7906	239	54	80	3.71 (3.30–4.15)
Octylisothiazolinone	0.1	7484	51	27	17	1.04 (0.82–1.30)
Decyl glucoside	5.0	9089	105	30	98	1.49 (1.25–1.76)
Lauryl glucoside	3.0	9414	107	12	111	1.26 (1.05–1.51)
*Evernia furfuracea* (tree moss)	1.0	3912	60	10	4	1.79 (1.40–2.26)
Sorbitan sesquioleate (SSO)	20	10 714	61	16	108	0.72 (0.57–0.90)
Sorbitan sesquioleate (SSO)	5.0	891	3	0	0	0.34 (0.07–0.98)
Sorbitan sesquioleate (SSO)	2.0	891	1	0	2	0.11 (0–0.62)
Parthenolide	0.1	3989	19	9	7	0.70 (0.47–1.01)
Black rubber mix	0.6	4335	20	8	8	0.65 (0.43–0.93)

*Note:* Conc., concentration in %, tested in petrolatum.

^a^ Compositae mix 2.5% (5.0%) pet. contains the following extracts and single compounds, respectively: *Anthemis nobilis* 0.6% (1.2%), *Chamomilla recutita* 0.6% (1.2%), *Achillea millefolium* 0.5% (1.0%), *Tanacetum vulgare* 0.5% (1.0%), *Arnica montana* 0.25% (0.5%), and parthenolide 0.05% (0.1%).

Among the 5147 patients tested with Compositae mix (CM) II 2.5% pet., 815 were also tested with parthenolide 0.1% pet. Concordance was high, with 7 patients positive and 804 negative to both preparations. One reacted only to parthenolide, and 3 only to CM II. Concordance quantified by an odds ratio (OR) was extremely high with 1876 (95% CI: 173.2–20318.2). CM II 5% pet. had also been used for patch testing, namely in 4755 patients, with 1.39 (1.08%–1.76%) positive reactions, that is, a nearly identical yield. No data were available to examine concomitant reactivity with parthenolide with the higher CM test concentration. The simultaneous testing of *N*‐Isopropyl‐*N*′‐phenyl‐*p*‐phenylenediamine (IPPD) and black rubber mix in 2124 patients enabled a comparison of positive reactions. These were seen in eight cases to both, in seven patients only to IPPD and—entirely symmetrically—also only to black rubber mix, while 2102 patients reacted to neither allergen (mixture).

Sorbitan sesquioleate (SSO) was tested in 3 different concentrations, albeit by only one department, whereas SSO 20% pet. was one of the most commonly tested additions (Table 2). The patient positive to SSO 2% also reacted to the two higher test concentrations, and the two patients reacting to SSO 5% also reacted to SSO 20% pet. Considering SSO being a dispersing agent in fragrance mix I (FM I), coupled reactivity between FM I and SSO 20% pet. was assessed. While most patients (*n* = 8145) did not react to either test allergen, 35 reacted positive to both. Discordant reactions were seen in terms of 35 patients reacting only to SSO, and 605 only to FM I (*p* < 0.00001, exact McNemar test). Notwithstanding such extreme asymmetry, the OR of 13.5 (95% CI: 8.4–21.7) indicates moderate association. Analyzing cross‐reactivity in more detail by considering the full scope of reactivity (Table S3), reactions classified as IR are altogether rare; with 16 seen to FM II and 16 to SSO. Further, it is interesting to note that (i) 12/16 IR reactions and (ii) 61/80?+ reactions to SSO occurred in patients reacting negatively to FM I, that is, the vast majority in both cases.

Concomitant reactivity between decyl and lauryl glucoside was assessed in the 2823 patients in whom individual data were available and both allergens had been patch tested: 26 patients reacted to both glucosides, 2759 to neither, 26 only to decyl glucoside and 12 only to lauryl glucoside (OR 229.9 (95% CI: 104.8–504.3)). Considering the present subgroup as representative, 0.43 (0.22%–0.74%) of consecutive patients would have been missed if relying on decyl glucoside for the screening of “glucosides contact allergy.”

Regarding *Everia furfuracea* (tree moss), concomitant reactivity was assessed toward FM I (as this contains *E. prunastri* extract) and toward colophonium, owing to (oxidized) resin acids usually contaminating tree moss, in those 2048 patients tested with tree moss (all tested with the two other, baseline series allergens). Concerning FM I, 20 reacted positively to both FM I and tree moss, 121 only to FM I, and 17 only to tree moss (OR 18.4 (95% CI: 9.4–36)). Regarding colophonium, 16 reacted positively to both colophonium and tree moss, 54 only to colophonium, and 21 only to tree moss (OR 27.6 (95% CI: 13.6–55.8)). In all, seven patients, that is, 0.34 (0.14%–0.7%), reacted only to tree moss, but not to FM I nor colophonium. A different perspective is to look at the positive predictive value (PPV) of being sensitized to tree moss if diagnosed allergic to FM I and colophonium, respectively. In this regard, 14.2 (8.9%–21.1%) of patients positive to FM I will be allergic to tree moss, while for colophonium the PPV is 22.9 (13.7%–34.5%).

Finally, three formaldehyde releasing preservatives considered as “recommended additions” to the EBS 2019 version have been included in the present analysis [3]. Cross‐reactivity between these and formaldehyde was examined. Among the 8148 patients patch tested with formaldehyde 1% aq. (i.e., half of the recommended patch test dose), (i) 150 were tested also with 2‐bromo‐2‐nitropropane‐1,3‐diol (Bronopol) 0.5% pet., not considered here owing to the small subsample size; (ii) 1381 with diazolidinyl urea 2% pet., yielding 25 (3.19%–65.09%) positive reactions also to formaldehyde among the 8 patients positive to diazolidinyl urea; and (iii) 1387 with imidazolidinyl urea 2% pet., yielding 6.67 (0.17%–31.95%) positive reactions also to formaldehyde among the 15 patients positive to diazolidinyl urea. Among the 5630 patients patch tested with formaldehyde 2% aq., (i) 1070 were tested also with 2‐bromo‐2‐nitropropane‐1,3‐diol 0.5% pet., yielding 0 (0%–52.18%) positive reactions also to formaldehyde among the five patients positive to 2‐bromo‐2‐nitropropane‐1,3‐diol; (ii) 2717 with diazolidinyl urea 2% pet., yielding 15 (3.21%–37.89%) positive reactions also to formaldehyde among the 20 patients positive to diazolidinyl urea; and (iii) 3357 with imidazolidinyl urea 2% pet., yielding 19.23 (6.55%–39.35%) positive reactions also to formaldehyde among the 26 patients positive to diazolidinyl urea. As imidazolidinyl urea has been removed from the “recommended additions” to the EBS 2023 version [4], co‐reactivity between this formaldehyde releaser and diazolidinyl urea remaining in the “recommended additions” was examined. Among the 4105 patients tested with both, 19 reacted to both, 9 only to diazolidinyl urea, and 19 only to imidazolidinyl urea; this difference was not statistically different (*p* = 0.09, McNemar test).

## 4 Discussion

The present report updates previous ESSCA report on the continually changing set of allergen preparations under scrutiny for possible inclusion into the EBS, most notably the previous analysis covering 2019/2020 [12]. Patch test results with the EBS obtained 2021/22 are presented and discussed in a separate publication [6]. Some general aspects are discussed in that paper which will not be reiterated here, for example, the lack of universally performed D7 readings and its consequences. In the following sections, important aspects concerning the selected allergens will be discussed allergen by allergen. These are summarized in Table S4. Benzisothiazolinone (BIT), sodium metabisulfite, and decyl glucoside have meanwhile been taken up into the currently used EBS, version 2023 [4].

### 4.1 Sodium Metabisulfite

Sodium metabisulfite, a marker for sulphite contact allergy, is still a common sensitizer with a fairly stable to even slightly higher sensitization prevalence of 3.49 (3.14%–3.88%) compared to the previous analysis (3.0 [2.58%–3.51%]) [12]. This aligns well with recent reports summarizing the increasing prevalence of sulphite contact allergy [13], also outside Europe, although sulphite contact allergy appears to be less prevalent in the US with 2.7% [14]. Clinical relevance, however, often remains difficult to establish (now only 39.5% vs. 58% previously), probably related to the very widespread and often hidden presence of sulphites. Nevertheless, recent investigative papers have again confirmed their potential relevance, mostly in cosmetics (incl. hair dyes) and pharmaceuticals (incl. injectable local anesthetics, transdermal therapeutic systems, and especially ophthalmic drugs) [15, 16, 17]. Food and alcoholic beverages are likely also under‐reported sulphite sources [18]. Moreover, newer sources of exposure concern gloves as cause of hand dermatitis [14] and clothing [13, 19]. Finally, although less common, novel occupational sources have been highlighted (e.g., in a baker) [20]. Another new finding is the potential relevance of sulphite contact allergy in patients with oral lichen planus [21]; however, clinical relevance of contact allergy to sodium metabisulfite is often difficult to elucidate in clinical practice, as also demonstrated by a relatively low %age of identified relevance in our results (Table 3).

**TABLE 3:** Clinical relevance of positive reactions in consecutively tested patients.

Allergen	Current relevance			Past relevance			% relevant
	NEC	occ.	non‐occ.	NEC	occ.	non‐occ.	(95% CI)
Sodium metabisulfite	63	1	25	9	0	2	39.5% (33.5%–45.8%)
2‐Bromo‐2‐nitropropane‐1,3‐diol	12	0	1	0	0	0	68.4% (43.4%–87.4%)
Diazolidinyl urea	11	2	4	2	0	0	47.5% (31.5%–63.9%)
Imidazolidinyl urea	5	4	4	1	0	1	32.6% (19.5%–48.0%)
Compositae mix (2.5%)	1	3	12	0	0	2	72.0% (50.6%–87.9%)
Compositae mix (5.0%)	28	2	3	3	3	0	59.1% (46.3%–71.0%)
Linalool hydroperoxides	154	13	181	12	1	5	75.2% (71.1%–78.9%)
Limonene hydroperoxides	154	6	167	7	0	3	74.7% (70.4%–78.7%)
Benzisothiazolinone	43	12	21	5	0	2	51.9% (43.8%–59.8%)
Octylisothiazolinone	14	1	4	1	0	1	40.4% (27.0%–54.9%)
Decyl glucoside	35	1	17	1	0	3	52.3% (42.5%–61.9%)
Lauryl glucoside	21	3	15	1	0	0	38.1% (28.8%–48.1%)
*Evernia furfuracea* (tree moss)	33	0	13	2	0	0	68.6% (56.4%–79.1%)
Sorbitan sesquioleate (SSO)	19	0	11	6	0	0	46.8% (35.3%–58.5%)
Parthenolide	11	2	4	2	0	1	66.7% (47.2%–82.7%)
Black rubber mix	1	3	2	1	0	0	25.0% (10.7%–44.9%)

*Note:* This was regularly documented in 11 541 patients for whom individual data on audit allergen test results were generally available (*N* = 11 610), that is, excluding aggregated results data from Spain, but including those from Leeds (UK), as relevance information (“current NEC” and “past NEC”) was included in these tabulated results. Thereby, the denominator for “% relevant” does not correspond to the results shown in Table 2.

Abbreviations: NEC, not elsewhere categorized; occ, occupational.

### 4.2 Benzisothiazolinone and Octylisothiazolinone

Compared to the previous analysis 2019/2020 [12], the prevalences of positive patch test reactions to BIT (3.71 (3.3%–4.15%)) and octylisothiazolinone (OIT) (1.04 (0.82%–1.3%)) remained high, if slightly lower than before and well above the threshold for inclusion in the EBS. However, clinical relevance was not always clear, especially for BIT. While BIT 0.1% pet. was included in the 2023 EBS version, OIT 0.1% pet. remained in the recommended additions [4]. Their use in cosmetics has never been allowed within the EU. Exposure to BIT and OIT is thus mainly related to non‐cosmetic products, such as paints, lacquers, detergents, metalworking fluids and adhesives, in which particularly BIT has been increasingly used [22]. This particular derivative has superseded MI in causing contact allergy in Europe [23]. Data from Belgium [24], Germany [25], UK [26], and Spain [27] have confirmed a remarkable increase in sensitization from this particular derivative, although relevance remains difficult to elucidate. In North America, the prevalence of BIT sensitization has recently increased from 7.3% in 2017–2018 to 10.4% in 2019–2020 [28, 29]. The higher prevalences might be explained by the use of BIT in cosmetics which is allowed in North America. Within the EU, BIT was re‐classified from a category 1 to a category 1A skin sensitizer in September 2024 (Regulation EU 2024/197 amending Regulation EC 1272/2008) and the specific concentration limit for labeling was slightly decreased from 0.05% to 0.036%, although this is still much higher than for MCI/MI and MI (0.0015%). Therefore, BIT is still an attractive preservative for industry. Regarding OIT, a recently published novel and remarkable source of consumer exposure concerns (synthetic) leather headphones [30, 31].

### 4.3 Sorbitan Sesquioleate

The emulsifier sorbitan sesquioleate (SSO) is a well‐known skin sensitizer in its own right [32], able to provoke relevant contact allergies due to its presence in for example, face cosmetics [33] and pharmaceuticals such as corticosteroid creams [34]. Moreover, it is a constituent of many patch test preparations in concentrations varying between 1% and 5%, including *Myroxylon pereirae* resin, decyl glucoside, 2‐hydroxyethyl methacrylate (HEMA) and, in particular, FM I [32], the latter regardless of the patch test supplier [35]. In the Information Network of Departments of Dermatology (IVDK), SSO 20% pet. has been part of the baseline series since 2015 and the prevalence of sensitization to SSO between 2016 and 2019 ranged from 0.6% to 0.9% in the participating departments in Germany, Austria and Switzerland [32], which is slightly higher than reported in Sweden (0.48%) [35], yet similar to what was observed here (0.72 (0.57%–0.9%)) and which actually exceeds the threshold for inclusion in the EBS. Independently, the presence of SSO in various patch test preparations must be considered when interpreting positive patch test reactions to for example, FM I. In the current data set, 35/640 (5.5%) of those with a positive patch test to FMI, were positive to SSO. In these patients, it is possible that they are not sensitized to fragrances, but to SSO, or both; in such patients, fragrance contact allergy may be difficult to confirm or to rule out [35]. Similarly, 5.5% of patients in the IVDK network with a positive patch test to FMI between 2016 and 2019 were concomitantly positive to SSO. Further, 35 patients reacted only to SSO, but not to FM I. This might be explained by the higher concentration of SSO in the 20% test preparation compared to the 5% concentration of SSO in FM I. Additionally, doubtful, irritant and potentially false‐positive reactions (i.e., usually showing a morphology of a + positive patch test) to SSO must be considered. Nevertheless, it was agreed upon to add SSO 20% pet., and its constituent sorbitan mono‐oleate 5% pet., as recommended additions to the 2023 EBS update until further evidence allowed a conclusion regarding the inclusion of SSO into the EBS [4]. In view of the presently found notable prevalence and not uncommon relevance 46.8% (35.3%–58.5%), and its aid in interpreting reactions to patch test preparations containing this emulsifier, inclusion appears reasonable and perhaps even necessary.

### 4.4 Compositae Allergy

Despite their natural origin often associated with being risk‐free by lay people, it is well known that Compositae allergens can induce contact allergies, particularly in high‐risk groups such as gardeners, florists, and agricultural workers [36]. Moreover, due to the increasing popularity of plant‐based cosmetic products [37], patch testing Compositae mix II (CM II), containing *Anthemis nobilis* , *Chamomilla recutita* , *Achillea millefolium* , *Tanacetum vulgare* , *Arnica montana* , and parthenolide, is probably increasingly important. Active sensitization to CM II remained undocumented in data from the IVDK network (Germany, Austria, Switzerland) at a test concentration of 5% pet [38]. Thus, concerns regarding active sensitization can probably be dismissed and testing CM II at 5% pet. for optimal screening has been suggested [4]. The present data include test results both with CM II 2.5% pet., yielding 1.4 (1.1%–1.76%) positives (Table 2), and other patients tested with CM 5% pet., which revealed an nearly identical sensitization prevalence of 1.39 (1.08%–1.76%). Importantly, the sesquiterpene lactone mix in the EBS alone is not sufficient to diagnose Compositae contact allergy [39]. Ideally, patch testing with suspected plants complements the work‐up [40]. Clinical relevance of contact allergy diagnosed with the lower concentration of the mix was slightly, but not significantly more common than with the higher concentration (72% vs. 59%, see Table 3).

### 4.5 Decyl and Lauryl Glucoside

Decyl glucoside clearly deserves its place in the current EBS [4], as the prevalence of sensitization is consistently high, currently 1.49 (1.25%–1.76%), very similar to the previous analysis with 1.43 (1.17%–1.73%) [12], and to recent Australian results where this glucoside derivative also merited its place in the baseline series [41]. Moreover, the share of patients in whom current relevance was identified, previously rather low at around 30% [12], appears to have considerably increased in the past years, amounting to more than 50% in the present analysis (Table 3). Relevant positive patch tests to glucosides have meanwhile also been documented in children [42], although concerns have been expressed regarding their potential irritancy in the patch test, especially in younger atopic children [43]. The additional yield of patch testing lauryl glucoside, no longer considered as audit allergen [4], was previously considered too limited to warrant inclusion in the EBS [12]. The present results, with 0.43 (0.22%–0.74%) of consecutive patients diagnosed additionally just with lauryl glucoside are still largely in line with this notion. A high degree of cross‐reactivity between glucosides is evident from the present results. In conclusion, the smaller molecule decyl glucoside appears to be a suitable screening marker for glucoside contact allergy, yet in selected patients separate testing to other glucosides may still be warranted [44].

### 4.6 Fragrances

Compared to the previous analysis [12], the results with the “recommended additions” terpene hydroperoxides are similar, with just slightly lower prevalences to linalool‐OOH 7.65 (7.09%–8.23%) tested 1% pet. and 4.77 (4.34%–5.23%) tested 0.5% pet., and similar prevalences with limonene‐OOH 6.78 (6.26%–7.32%) tested 0.3% pet. and 5.41 (4.89%–5.96%) tested 0.2% pet. The present results do not provide additional new evidence supporting a decision to recommend inclusion of one or the other respective patch test concentration into the EBS, as discussed in detail in a recent review [45]. *Evernia furfuracea* (tree moss) 1% pet. yielded 1.79 (1.4%–2.26%) positive reactions, similar to the previous round (1.43 (1.08%–1.86%) positive reactions) [12]. The additional yield of 0.34 (0.14%–0.7%) patients which are missed still does not appear to justify inclusion into the EBS. The cross‐reactivity owing to the presence of constituents common between *E. prunastri* and *E. furfuracea* on the one hand, and (oxidized) resin acids found in pine (bark) extracts and colophonium on the other is well‐known. However, the “diagnostic performance” of using either FM I or colophonium as screener for contact allergy to tree moss is limited, in view of the rather modest PPVs.

### 4.7 Formaldehyde Releasing Preservatives

The present results with the three formaldehyde releasers are in a similar range, below 1% positive reactions in consecutive patients, as observed before [12]. Moreover, confirming a previous in‐depth analysis addressing the ability to detect contact allergy to these releasers by using formaldehyde 2% aq. as screener [46], the limited co‐reactivity has been confirmed. This is also true if formaldehyde 1% aq. is used in lieu of 2% aq., mostly owing to non‐availability of an authorized patch test preparation for 2% aq [47].

### 4.8 Black Rubber Mix

Black rubber mix (BRM) is not a “new” test substance, but has been tested for example, at St. John's Institute, London, since 1971, yielding 0.8% positive reactions in male and 0.3% in female patients patch tested 1971–1976 [48]. However, in 1992 it was replaced by IPPD [48], also in the EBS which had been first developed at the time [49]. This change was introduced by the patch test material producer Hermal/Trolab due to the unavailability of two components (*N*‐phenyl‐*N*′‐cyclohexyl‐*p*‐phenylenediamine (CPPD) and *N*,*N*′‐diphenyl‐*p*‐phenylenediamine (DPPD), each with a concentration of 0.25%) of this mix in sufficient chemical purity. As IPPD, tested 0.1% both in the mix and single, was the allergen yielding the highest number of positive reactions and also often cross‐reacting with CPPD and DPPD, resp., IPPD replaced BRM. However, diagnostic sensitivity was slightly impaired, as 10% of patients allergic to CPPD and/or DPPD did not react to IPPD when tested with individual components [48]. At present, both CPPD and DPPD are available in the required purity, and a number of (inter)national baseline series include BRM, while the EBS and most European national series still contain IPPD only. Discussions are currently underway regarding the possible reinstatement of BRM to the EBS after the 30‐year absence. The share of 0.65 (0.43%–0.93%) positive reactions seen in the present analysis, 25% (10.7%–44.9%) of these considered relevant, does not seem to solve the discussion. The current ESSCA results with the EBS document 0.56 (0.45%–0.68%) positive reactions to IPPD 0.1% pet. and 0.89 (0.57%–1.32%) positives to BRM as included in the TRUE‐Test in 2702 Spanish patients (no data on relevance) [6]. During the reporting period 2019/20, 0.88 (0.61%–1.25%) positive reactions to BRM contained in the TRUE‐Test were found (BRM 0.6% pet. not having been reported) and 0.79 (0.66%–0.94%) to IPPD 0.1% pet [50]. Further studies comparing the comparative yield of positive reactions and clinical relevance BRM and IPPD are needed to assess the benefit of replacing IPPD with BRM in the EBS.

## 5 Conclusion

The current results will, together with other published evidence, inform the decision process concerning possible adaptations of the EBS. The ESSCA working group and the EBS task force of the ESCD, and the GEIDAC network thus contribute to updating the EBS in a timely fashion, aiming at a high level of diagnostic standards and patient care.

## Author Contributions

**Wolfgang Uter:** conceptualization, methodology, software, formal analysis, visualization, project administration, writing – original draft, writing – review and editing. **Stephen Mark Wilkinson:** conceptualization, data curation, validation, funding acquisition, writing – original draft, writing – review and editing, investigation. **Andrea Bauer:** data curation, validation, investigation, writing – review and editing. **Leopoldo Borrego:** conceptualization, data curation, funding acquisition, investigation, methodology, software, validation, writing – original draft, writing – review and editing. **Richard Brans:** writing – original draft, writing – review and editing, data curation, validation, investigation, conceptualization. **Timo Buhl:** data curation, writing – original draft, writing – review and editing, investigation, validation. **Heinrich Dickel:** writing – original draft, writing – review and editing, investigation, validation, data curation. **Rosella Gallo:** data curation, writing – review and editing, investigation, validation. **Ana Giménez Arnau:** writing – original draft, writing – review and editing, data curation, investigation, validation. **Margarida Gonçalo:** data curation, investigation, validation, writing – original draft, writing – review and editing. **Dimitra Koumaki:** data curation, investigation, validation, writing – original draft, writing – review and editing. **Alexander A. Navarini:** writing – review and editing, data curation, investigation, validation. **Cataldo Patruno:** data curation, validation, writing – review and editing. **Györgyi Pónyai:** data curation, investigation, validation, writing – review and editing. **Thomas Rustemeyer:** writing – review and editing, data curation, investigation. **Radoslaw Spiewak:** writing – review and editing, writing – original draft, investigation, validation, data curation. **Luca Stingeni:** data curation, conceptualization, validation, writing – original draft, writing – review and editing. **Skaidra Valiukevičienė:** data curation, investigation, validation, writing – review and editing. **Marie‐L. A. Schuttelaar:** conceptualization, writing – original draft, writing – review and editing, project administration, validation, investigation, data curation. **Olivier Aerts:** writing – original draft, writing – review and editing, data curation, investigation, validation, supervision, conceptualization.

## Funding

This work was supported by the European Academy of Dermatology and Venereology EADV Grant PPRC‐2018‐8. The REIDAC project is sponsored by the Spanish Academy of Dermatology and Venereology (Fundación Piel Sana) and has received funding from the Spanish Medicines and Health Products Agency (Agencia Española de Medicamentos y Productos Sanitarios; available at https://www.boe.es/boe/dias/2023/12/15/pdfs/BOE‐A‐2023‐25482.pdf), Sanofi‐Aventis, and LEO Pharma. The REIDAC sponsors were not involved in the proposal, preparation, design, data extraction, analysis, interpretation, drafting, editing, review, approval, or logistical support associated with this manuscript.

## Conflicts of Interest

W.U. receives research funds directed to the department from the cosmetic industry association IFRA. O.A. is investigator, consultant and/or speaker for LEO Pharma, Abbvie, Sanofi, L'Oréal/La Roche Posay, Novartis, Amgen, and Bioderma/NAOS. A.B. has been a speaker/advisor/investigator and/or received research funding from AbbVie, Almirall, Amgen, AstraZeneca, Biocryst, Biofrontera, Celldex, CSL Behring, Eli Lilly, Escient, Galderma, Genentech, Gilead, Incyte, Janssen, Jasper, Kalvista, LEO Pharma A/S, L'Oréal, Novartis, Otsuka, Pfizer, Pierre Fabre, Pharvaris, Regeneron, Sanofi and Takeda. R.B. served as advisor and speaker for LEO Pharma. H.D. is an investigator, consultant and/or speaker for Almirall Hermal, Stallergenes, LEO Pharma, Sanofi‐Aventis and Novartis Pharma. A.G.A. is or recently has been a speaker and/or advisor for and/or has received research funding from Almirall, Amgen, AstraZeneca, Avene, Blue ‐Print, Celldex, Celltrium, Escient Pharmaceutials, Genentech, GSK, Harmonic Bio, Incyte, Instituto Carlos III‐ FEDER, Jaspers, LEO Pharma, Menarini, Mitsubishi Tanabe Pharma, Noucor, Novartis, Sanofi–Regeneron, Septerna, Servier, Thermo Fisher Scientific, Uriach Pharma. R.S. is part‐time employee and shareholder at the Instytut Dermatologii, Krakow, Poland, received lecture honoraria from the Polish Societies of Allergology and of Dermatology, Forum Media, MedicaExpert, Ginemedica, LEO Pharma and Chiesi. M.‐L.A.S. is an advisor, consultant, speaker and/or investigator for AbbVie, Amgen, Pfizer, LEO Pharma, Regeneron Pharmaceuticals, Sanofi Genzyme, Incyte, Galderma, International Fragrance Association. S.V. has been a speaker/advisor/from AbbVie, Almirall, Amgen, Eli Lilly, Janssen, LEO Pharma, L'Oréal, Novartis, Pfizer, and Sanofi. S.M.W. has received travel reimbursement to attend meetings with the cosmetic industry. The other authors declare no conflicts of interest.

## Supporting information


**Table S1:** Contribution by department and country, respectively.
**Table S2:** MOAHLFA(P) index [11] per country including those patients patch tested with (at least one) of the audit allergens in the ESSCA network, 2021/22.
**Table S3:** Cross‐tabulation of patch test results with fragrance mix (FM) I sorbitan sesquioleate (SSO) 20% pet. in *n* = 8820 patients for whom individual data were provided and who had been tested with both allergen preparations, 2021/22.
**Table S4:** Summary of important allergen characteristics and main findings.

## Data Availability

The data that support the findings of this study are not available owing to data protection requirements.
