# Supplementary material for: Patch Test Results With Additions to the European Baseline Series, 2021/22—Joint European Results of the ESSCAA and the EBSB Working Groups of the ESCD, and the GEIDACC
Source: Contact Dermatitis. 2026 May 14;95(3):276–85. doi: 10.1111/cod.70183 (PMC13441338; doi:10.1111/cod.70183)
Supplement: Supplementary file 1 [file COD-95-276-s001.docx]

**Online supplemental Table S1**: Contribution by department and country, respectively. Additional contributions are from the “Grupo Español de Investigación en Dermatitis de Contacto y Alergia Cutánea” (GEIDAC):^5^ additional 3872 patients.

| Country | City | 2021 tested | 2022 tested |
| --- | --- | --- | --- |
| Austria | Graz | 95 | 89 |
| Belgium | Antwerp | 400 | 491 |
| Switzerland | Basel | 131 | 115 |
| Switzerland | Bern | 285 | 312 |
| Switzerland | Aarau | 108 | 92 |
| Switzerland | Lausanne/Epalignes | 24 | 10 |
| Germany | Dortmund | 35 | 20 |
| Germany | Göttingen | 74 | 79 |
| Germany | Kiel | 181 | 75 |
| Germany | Dresden | 201 | 180 |
| Germany | Jena | 141 | 146 |
| Germany | Mainz | 116 | 116 |
| Germany | Osnabrück | 309 | 292 |
| Germany | Erlangen | 233 | 190 |
| Germany | Heidelberg | 77 | 65 |
| Germany | Hannover | 139 | 121 |
| Germany | Bochum | 283 | 335 |
| Greece | Athens | 0 | 346 |
| Greece | Heraklion, Crete | 54 | 101 |
| Hungary | Budapest | 247 | 262 |
| Italy | Napoli | 292 | 331 |
| Italy | Genova | 170 | 0 |
| Italy | Perugia | 376 | 175 |
| Lithuania | Kaunas | 310 | 391 |
| The Netherlands | Groningen | 271 | 263 |
| The Netherlands | Amsterdam VU | 702 | 693 |
| Poland | Krakow | 41 | 45 |
| Portugal | Coimbra | 272 | 252 |
| United Kingdom | Middlesbrough | 193 | 0 |
| United Kingdom | Leicester | 83 | 180 |
| United Kingdom | Leeds | 869 | 1008 |

**Online supplemental Table S2**: MOAHLFA(P) index^11^ per country including those patients patch tested with (at least one) of the audit allergens in the ESSCA network, 2021/22.

| Country | % male | % occup. | % AD | % hand derm. | % leg derm. | %face derm. | % age 40+ | % baseline pos. |
| --- | --- | --- | --- | --- | --- | --- | --- | --- |
| AT | 20.1 | 15.2 | 20.7 | 23.4 | 4.3 | 15.8 | 63.0 | 53.8 |
| BE | 29.2 | 10.1 | 37.7 | 19.6 | 7.0 | 25.7 | 56.9 | 51.1 |
| CH | 34.8 | 15.8 | 23.7 | 26.3 | 6.9 | 16.2 | 66.0 | 52.5 |
| DE | 36.6 | 34.6 | 30.5 | 45.7 | 4.2 | 9.2 | 70.0 | 36.6 |
| ES | 31.2 | 8.5 | 19.0 | 31.7 | 4.8 | 21.7 | 66.0 | 36.1 |
| GR | 29.9 | 27.9 | 18.2 | 33.3 | 5.2 | 23.4 | 60.1 | 66.7 |
| HU | 24.2 | 0.0 | 15.7 | 39.7 | 2.8 | 20.4 | 63.3 | 50.7 |
| IT | 27.5 | 5.2 | 17.1 | 23.9 | 1.3 | 3.5 | 58.9 | 63.8 |
| LT | 23.4 | 22.0 | 7.7 | 30.4 | 7.3 | 25.1 | 46.6 | 30.5 |
| NL | 30.5 | 11.9 | 33.9 | 13.0 | 2.8 | 5.9 | 61.2 | 66.2 |
| PL | 25.9 | 0.0 | 20.0 | 0.0 | 0.0 | 0.0 | 31.8 | 60.0 |
| PT | 34.7 | 21.9 | 15.6 | 33.0 | 3.1 | 18.3 | 65.6 | 44.5 |
| UK | 29.8 | 5.1 | 49.1 | 26.0 | 4.5 | 37.6 | 46.8 | 49.7 |

occup., occupationally caused dermatitis; AD, atopic dermatitis; derm., dermatitis; baseline pos., positive to at least one of the baseline series allergens

**Online supplemental Table S3**: Cross-tabulation of patch test results with fragrance mix (FM) I in rows and sorbitan sesquioleate (SSO) 20% pet. in columns by detailed degree of reaction in n=8820 patients for whom individual data were provided and who had been tested with both allergen preparations, 2021/22.

|  | neg. | IR | ?+ | + | ++ | +++ |
| --- | --- | --- | --- | --- | --- | --- |
| neg. | 7862 | 12 | 61 | 23 | 7 | 0 |
| IR | 15 | 0 | 0 | 1 | 0 | 0 |
| ?+ | 187 | 0 | 8 | 4 | 0 | 0 |
| + | 383 | 4 | 10 | 23 | 2 | 0 |
| ++ | 169 | 0 | 1 | 3 | 1 | 1 |
| +++ | 38 | 0 | 0 | 0 | 4 | 1 |

**Online supplemental Table S4**: Summary of important allergen characteristics and main findings.

| **Sensitizer** | **Main exposure sources** | **Tested (Conc.)** | **Positive**  **(%)** | **Relevance**  **(%)** |
| --- | --- | --- | --- | --- |
|  | *Haptens introduced into EBS 2023* |  |  |  |
| Benzisothiazolinone | Biocide used in interior paints and other finishing materials, household (cleaning) products, laundry detergents/softeners. | 7906 | 3.71 | 51.9 |
| Sodium metabisulfite | Preservative, antioxidant and acidity regulator common in cosmetics, food products and pharmaceuticals, and e.g. textile, paper, metal, and construction industry. | 9703 | 3.49 | 39.5 |
| Decyl glucoside | Detergent/emulsifier present in many cosmetic products | 9089 | 1.49 | 52.3 |
|  | *Recommended additions to EBS 2023* |  |  |  |
| Linalool hydroperoxides | (Aut)oxidation products of linalool – a fragrance widely used in most cosmetic products, cleaning and laundry products, and others. | 8566 (1%) | 7.65 | 75.2 |
|  |  | 9091 (0.5%) | 4.77 |  |
| Limonene hydroperoxides | (Aut-)oxidation products of limonene – a fragrance widely used in most cosmetic products, cleaning and laundry products, and others. In (citrus) essential oils. | 8911 (0.3%) | 6.78 | 74.7% |
|  |  | 7046 (0.2%) | 5.41 |  |
| Compositae mix II | Extracts of important sensitizing plants from the Compositae family. | 4755 (5%) | 1.40 | 62.6 |
|  |  | 5147 (2.5%) | 1.39 |  |
| Octylisothiazolinone | Preservative in paints/coatings, wood preservatives, machinery coolants and cutting fluids, textile and leather industries. | 7484 | 1.04 | 40.4 |
| Sorbitan sesquioleate | Emulsifier in cosmetic and household products, pharmaceuticals for external use (ointments, creams), veterinary products. | 10714 (20%) | 0.72 | 46.8 |
|  |  | 891 (5%) | 0.34 |  |
|  |  | 891 (2%) | 0.11 |  |
| Diazolidinyl urea | Preservative (formaldehyde releaser) used in cosmetic products. | 8972 | 0.58 | 47.5 |
| 2-Bromo-2-nitropropane-1,3-diol | Biocide used in household products, cosmetic products and pharmaceuticals. Industrial applications include e.g. coolants, paints, detergents, and agricultural seed processing. | 6441 | 0.53 | 68.4 |
|  | *Other haptens included in the present study* |  |  |  |
| *Evernia furfuracea* | Fragrance in cosmetics, essential oil also used in aromatherapy (tree moss). | 3912 | 1.79 | 68.6 |
| Lauryl glucoside | Detergent/emulsifier present in many cosmetic products. | 9414 | 1.26 | 38.1 |
| Parthenolide | Natural compound in some Compositae plants, part of Compositae Mix II | 3989 | 0.70 | 66.7 |
| Black rubber mix | Three antioxidants/antiozonants in black rubber. | 4335 | 0.65 | 25.0 |
| Imidazolidinyl urea | Preservative (formaldehyde releaser) in cosmetic products | 9618 | 0.59 | 32.6 |
